# Is Chelation Therapy a Potential Treatment for Parkinson’s Disease?

**DOI:** 10.3390/ijms22073338

**Published:** 2021-03-24

**Authors:** Roberta J. Ward, David T. Dexter, Antonio Martin-Bastida, Robert R. Crichton

**Affiliations:** 1 Centre for Neurodegeneration and Neuroinflammation, Division of Brain Sciences, Department of Medicine, Imperial College, London W12 0NN, UK; ddexter@parkinsons.org.uk; 2 Parkinson’s UK. 15 Vauxhall Bridge Road, London SW1V 1EJ, UK; 3 Department of Neurology and Neurosciences, Clínica Universidad de Navarra, Pamplona, 28027 Madrid, Spain; amartin@unav.es; 4 Faculty of Science, Universite de Catholique de Louvain, 1348 Louvain-la-Neuve, Belgium; robert.crichton@uclouvain.be

**Keywords:** iron, chelators, Parkinson’s disease, neuroinflammation

## Abstract

Iron loading in some brain regions occurs in Parkinson’s Disease (PD), and it has been considered that its removal by iron chelators could be an appropriate therapeutic approach. Since neuroinflammation with microgliosis is also a common feature of PD, it is possible that iron is sequestered within cells as a result of the “anaemia of chronic disease” and remains unavailable to the chelator. In this review, the extent of neuroinflammation in PD is discussed together with the role played by glia cells, specifically microglia and astrocytes, in controlling iron metabolism during inflammation, together with the results of MRI studies. The current use of chelators in clinical medicine is presented together with a discussion of two clinical trials of PD patients where an iron chelator was administered and showed encouraging results. It is proposed that the use of anti-inflammatory drugs combined with an iron chelator might be a better approach to increase chelator efficacy.

## 1. Perspective and Introduction

In recent clinical trials, iron chelation therapy has been shown to open the way to a treatment of Parkinson’s disease, PD, which potentially slows the disease progression. However, since PD is a multifactorial disease, a “single target drug” such as an iron chelator may not be sufficient on its own to induce complete neuroprotection. In this context, the design and clinical evaluation of multifunctional drugs that combine iron chelation with other protective properties may be required.

The long march of increasing human life expectancy continues unabated, and with it the age profile of the world’s population. On average, life expectancy increases by 2–4 years every decade: in the 1950s, the average life expectancy was 45; by the 1980s, it was 60, and has now attained 75 years [1]. For the first time, in 2020, the over 60s will outnumber the number of children under the age of 5 [2]. However, with increasing age, disorders of the central nervous system will become more prevalent and impact on both the quality and the longevity of the elderly. Many of these neurological disorders, including Alzheimer’s and Parkinson’s diseases, appear to be related to focal accumulation of iron in specific regions of the brain [3,4,5].

## 2. Parkinson’s Disease

Parkinson’s Disease (PD) is the second most common form of motor system degeneration. PD affects approximately 1% of the population over the age of 60 and about 4% of those above 85 years old. PD is clinically heterogeneous. Symptoms of the disease include bradykinesia (slowness of movement), rigidity, resting tremor, postural instability, as well as non-motor symptoms such as sleep disturbance, constipation, dysarthria, dysphonia, and dysphagia. The decreased levels of dopamine in the striatum are caused by the progressive loss of dopamine-containing neurons in the substantia nigra pars compacta, SN, which results in decreased levels of dopamine in the striatum. When the loss of striatal dopamine reaches 60–70%, the motor symptoms of PD manifest. Insoluble protein inclusions in neurons (Lewy bodies), which are predominantly aggregates of misfolded α-synuclein (αSyn), together with the loss of dopaminergic (DA) neurons in the brain are the main neuropathological hallmarks of PD. The Lewy bodies and protein deposits are present initially in the dorsal motor nucleus (DMN) of the vagus nerve in the lower brain stem and then spread to the SN and thereafter to other brain regions with disease progression. The biological explanation for αSyn aggregation and neuronal loss remains unknown, although recent studies have suggested that the gut may play an important initiating role via the vagal link to the DMN [6].

### 2.1. Iron Loading in PD

One of the hallmark features of PD brains is the increased accumulation of iron in the SN [7], with a smaller accumulation of iron in the red nuclei, globus pallidus, and cortex of PD patients. With increased disease severity, the total iron content of the SN increases, which correlates with motor abnormalities [8] as well as microgliosis [9]. Iron deposits are present within the neurons and glia of the SN, putamen, and globus pallidus, with an increase of ferritin-loaded microglia cells in the SN [10]. The increase of iron in the SN of PD patients is associated with increased iron loading of ferritin and neuromelanin [10,11]. In addition, an increased expression of divalent metal transporter 1 may contribute to PD pathogenesis via its capacity to transport ferrous iron [12]. Dexter et al. [7,13] were one of the first groups to demonstrate that the neurodegenerative process in PD is associated with elevated iron levels in the SN. Various factors have been suggested for such iron loading: increased permeability or dysfunction at the blood–brain barrier (BBB), increase of lactoferrin receptors in neurons and micro vessels, increased DMT1 expression in dopamine neurons, mutations in genes relevant to iron transport and binding [3]. However, in recent studies, we have shown that an increased pro-inflammatory state may be significant, the increasing iron deposition being associated with the degree of microgliosis [9], thereby implicating the anaemia of chronic disease in contributing to the increased iron loading in the brain.

### 2.2. Non-Invasive Evaluation of Iron Loading

Recently, several Magnetic Resonance Imaging (MRI) imaging modalities have been utilised to noninvasively detect brain iron loading. Transverse relaxation (T2*) is obtained by angling the longitudinal relaxation (T1*) into the transverse plane by using a pulse of radiofrequency. The speed of relaxation is based on the firmness of the tissue or inhomogeneities that limit the water free movements, generating a reversible field dephasing effect. Relaxometry techniques present an important limitation, as T2* and its inverse, R2*, are highly influenced by the brain water content, water thereby limiting the value of iron-related structural pathology [14].

**Figure 1:**
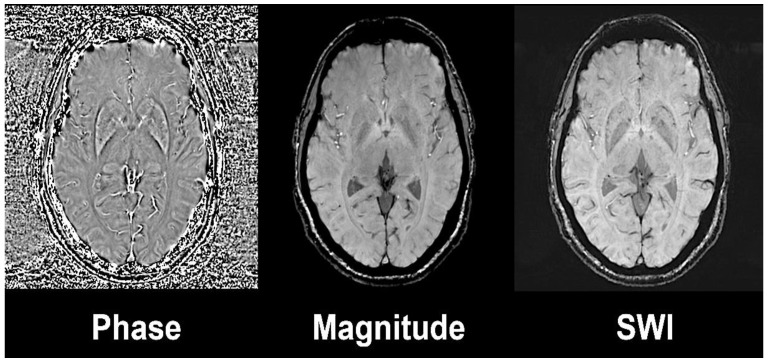
Susceptibility weighted image (left) is the result of the combination of high-pass filtered phase (left) and magnitude image (central). Axial slices at the level of basal ganglia on a 3 Tesla Siemens Magnetom Trio system with 32-channel phased-array head coil. Reprinted with the permission of [15].

Susceptibility weighted imaging (SWI) exploits the magnetic susceptibility in a tissue that differs from the susceptibility of neighbouring tissues [14]. Magnetic susceptibility is described as the magnetic response of a substance or tissue when an external radiofrequency pulse is applied. SWI combines a phase and a magnitude image by applying a gradient echo (GRE) with high magnetic field (Figure 1). The phase sequences represent an average of magnetic field protons that directly depend on the local susceptibility of the tissues. Paramagnetic substances, namely hemosiderin and ferritin, elevate the magnetic field, so there is a positive phase shift relative to the surrounding tissue, while diamagnetic substances, such as calcium, will cause a negative phase shift.

Recent developments in iron magnetic imaging led to Quantitative Susceptibility Mapping (QSM). This technique uses phase images solving the magnetic susceptibility inverse source problem, generating a three-dimensional susceptibility distribution [16].

Ferritin and to a lesser degree, hemosiderin, are considered the forms of non-haemic iron present in brain that could potentially affect the MR contrast [17]. At least one-third of brain iron is found in the form of ferritin, although this concentration is higher in grey matter and deep brain nuclei such as globus pallidus and SN [18]. Brain transferrin concentrations are very low when compared to ferritin, and together with other iron forms such as the labile-free iron pool are present at too low a concentration to affect the MR signal.

Ferritin is the primary storage iron protein, containing several thousand atoms of iron contained within mineralised ferric oxyhydroxide nanoparticles within the core of the protein. These nanoparticles embedded in ferritin act as magnetic super-units; hence, their magnetic property is known as superparamagnetism, manifested, by analogy to classical paramagnetism, as proportional to the reciprocal of the absolute temperature (1/T) [19]. Furthermore, hemosiderin is also superparamagnetic, as its particles are larger than ferritin. Both iron storage proteins, ferritin and hemosiderin, are associated with T2* and T2 shortening.

Several studies using MRI iron-sensitive sequences in PD are described in the literature, mainly using relaxometry techniques with a fewer number using SWI and QSM. Most of the imaging studies showed significant elevated nigral iron accumulation in PD when compared to controls [20,21,22,23]. Importantly, nigral iron accumulation is associated with disease severity as assessed by Unified Parkinson’s Disease Rating Scale (UPDRS)-III motor score [23] and to a lesser degree with disease duration [24] thus being considered as a potential biomarker of clinical severity in PD.

On the other hand, results pertaining to striatal iron accumulation in PD are heterogeneous, with several studies reporting increases [23], decreases [25], or normal values in PD [21] inconsistent with associations with clinical severity. A recent meta-analysis examining post-mortem and in vivo imaging studies including relaxometry and SWI techniques [26] concluded that imaging techniques may not authentically reflect iron changes in striatal regions other than in SN.

Neuromelanin (NM) is a dense, insoluble pigment present in several neuronal subtypes, but specifically in the dopaminergic neurons of SN and noradrenergic neurons of locus coeruleus (LC) [27] at high concentrations. NM has strong affinity for metals, i.e., iron, copper, lead, and zinc [28]. Iron is sequestered by the melanic portion of NM and maintained in a redox inactive form [28]; hence, NM could act as a “black hole” chelating redox-active metals. NM binds iron in two different high- and low-affinity sites. High-affinity sites of NM bind most of the available iron, although under iron overload conditions, these sites of NM become saturated, and the iron will also bind to the low-affinity sites [29]. When iron is bound to the low-affinity sites of NM, iron is sequestered in its reactive form and could potentially have an adverse role by promoting redox reactions and oxidative stress.

**Figure 2:**
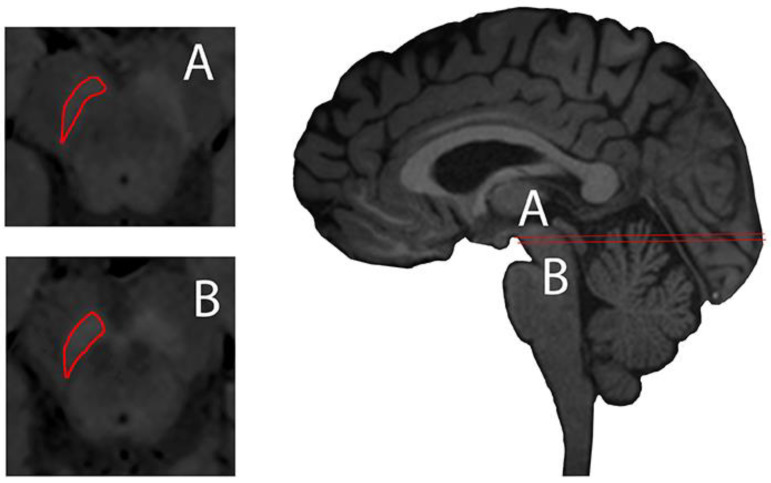
Substantia nigra delineated (red) axial plane of neuromelanin-sensitive Magnetic Resonance (MR) imaging (left, A and B) and sagittal midline plane of a 3D T1-weighted MPRAGE illustrating axial planes of ROI delineation. Images taken on a 3 Tesla Siemens Magnetom Trio system with a 32-channel phased-array head coil. Reprinted with the permission of [15].

NM-sensitive magnetic imaging was recently described using high-resolution T1 weighted (T1w) with Fast Spin Echo (FSE) on 3 Tesla MR, describing the visualisation of NM contrast in midbrain and brain tissue of the NM-pigmented nuclei [30] (Figure 2). The contrast obtained in neuromelanin-sensitive MRI seems to be directly related to the reduction of T1 from the melanin compounds [31].

A few imaging studies using NM-sensitive MR in PD have been undertaken with the common finding of reduction of NM contrast in the SN in PD subjects when compared to controls [31,32,33], which in turn associate with disease duration [34]. Results pertaining to LC depigmentation are heterogeneous, with several studies reporting reductions [31] or normal values in PD [32].

Furthermore, NM-sensitive imaging allows investigation of the relationship between nigral depigmentation and the dopaminergic striatal transporter (DAT) with positron emission imaging (PET) in relation with motor clinical laterality in PD [34].

### 2.3. Neuroinflammation in PD

The extensive microglia activation was identified in the SN of autopsied PD patients over 30 years ago [35]. These microglia remain chronically active and contribute to the degeneration of dopaminergic neurons. Such microgliosis could induce iron accumulation within the microglia [9]. The self-perpetuation of the inflammatory process is caused by the sustained release of misfolded α-synuclein aggregates, neuromelanin, ATP, and matrix metalloproteinase-3 (MMP-3) from dying dopaminergic neurons [36,37,38]. The release of such pro-inflammatory neuronal factors results in the production of a multitude of signal cascades, e.g., the release of pro-inflammatory enzymes, inducible nitric oxide synthase (iNOS), cyclooxygenase 2 (COX-2) [39,40], and a macrophagic protein, CD68, which is usually associated with phagocytic activity [41,42,43], pro-inflammatory cytokines, such as interleukin-1 beta, IL-1β, interleukin-2, IL-2, interleukin-6, IL-6, epidermal growth factor, EGF, transforming growth factor alpha, TGF-α, and transforming growth factor beta TGF-β, in the striatum [44] and tumor necrosis factor, TNF-α upregulation in nigral microglia [45], all of which may alter iron homeostasis. Both TNFα and transforming growth factor beta 1 (TGF-α1) may upregulate divalent metal transporter 1, DMT1 and downregulate ferroportin (FPN) in microglia [46], thereby promoting iron accumulation in the microglia [47], which might be instrumental in iron-induced dopaminergic degeneration in the SN. Furthermore, Il-β and TNFα may activate iron responsive element 1, IRP1, upregulating DMT1 (with the iron-responsive element, IRE), and transferrin receptor 1, TfR1, and downregulating ferroportin, FPN, the iron export protein, in neurons [48] with the net result of further increasing the iron content within these cells.

In a recent study, we demonstrated extensive microgliosis in the SN of postmortem PD patients, although this was similar in the patients when divided according to Braak criteria. A wide heterogeneity of microglial cells was present, ranging from resting ramified cells, long ramified processes with small cell bodies, to spherical activated cells with shorter processes and large cell bodies. Iron staining revealed an association between the intensity of microgliosis and iron deposition [9].

Astrocytes are the most abundant cells in the brain and maintain the homeostasis of the brain microenvironment. Astrocytes are involved in the formation of the blood–brain barrier (BBB); some 95% of the capillary surface is covered by end feet of astrocytes, and they play an important role in iron transport across the BBB and the maintenance of brain homeostasis, possibly via DMT 1 [49]. When activated, they possibly exist in two different types of reactive astrocytes termed A1 and A2. A1 reactive astrocytes principally induced by IL-1β and TNF-α secreted by activated neuroinflammatory microglia lose their ability to promote neuronal survival, synaptogenesis, and phagocytosis, [50]. In turn, they acquire a new neurotoxic function, which induces the death of neurons and oligodendrocytes and ultimately contributes to the cell death of DA neurons in the SN during neurodegeneration [50,51,52]. It is uncertain whether astrocytes produce pro-inflammatory mediators; they may only express chemokines. The zinc transporter Zip14 and the resident transient receptor potential channel may play an important role in non-transferrin bound iron, NTBI uptake [53,54]. Astrocytes can store ferritin and release iron via FPN. Caeruloplasmin may also play an important role by oxidising Fe^2+^ to Fe^3+^ and promote FPN-mediated iron release [54]. Astrocytes may affect iron metabolism and the survival of neurons by their ability to release neurotrophic factors such as brain-derived neurotrophic factor, BDNF, and glial line-derived neurotrophic factor, GDNF [54]. Inflammation will induce hepcidin expression in the brain, probably from astrocytes that are activated by microglia, via IL-6 signaling, to stimulate astrocytes to release hepcidin. In turn, this will signal to neurons and microglia, via hepcidin, to prevent their iron release [55].

### 2.4. Non-Invasive Evaluation of Neuroinflammation

The translocator protein 18 kDa (TSPO) is a five-transmembrane domain protein that is present in the outer mitochondrial membrane. In the healthy CNS, TSPO is constitutively expressed by multiple cell types, i.e., glia and neurons, but only at low levels. However, during inflammatory responses, TSPO is substantially upregulated, predominantly, in microglial cells [56]. [^11^C] (R)-PK11195 positron emission tomography images of activated microglia are shown to have increased binding in the pons, basal ganglia, and frontal and temporal cortical regions of PD patients [57]. Interestingly, the [^11^C] (R)-PK11195 signal remained stable over 2 years in studies of PD patients, the levels of microglial activation not correlating with clinical severity or putamen [18F]-dopa uptake. The authors suggested that the absence of any change in microglia activity might indicate that the disease is driven by their continuous release of cytokines. Ouchi et al. [58] reported that microglial activation and dopaminergic terminal loss declined in parallel in the affected nigrostriatal pathway in early PD, as measured with the DAT radiopharmaceutical [^11^C]-2-β-carbomethoxy-3β-(4-fluorophenyl) tropane [^11^C]-CFT. Studies that have investigated whether drugs, i.e., minocycline and celecoxib, could modulate the activation of microglial cells have yielded inconclusive results [59,60].

In the last few years, a second generation of TSPO tracers have been introduced that have improved microglial activation imaging. These new tracers, including [^18^F]-FEPPA, [^11^C]-PBR28, [^11^C]-DPA713, or [^18^F]-DPA714, which are more sensitive to the polymorphism rs6971, are located in the TSPO gene that is responsible for the different affinity patterns [61]. Increased microglial activations have been found in nigrostriatal pathways including midbrain and putamen as well as frontal cortex; however, no significant associations with clinical measures were demonstrated with second-generation TSPO tracers [62].

Astrocyte numbers increase during aging and are involved in glial fibrillary acidic protein (GFAP) regulation and neuroinflammation [63,64]. Astrocytosis can be imaged by PET using [^11^C]-Deuterium-Deprenyl [65], which is a radiopharmaceutical that targets monoamine oxidase type B (MAOB), or by the imidazoline-2 receptor radioligand [^11^C]-2-(4,5-dihydro-1H-imidazol-2-yl)-1-methyl-1H-indole ([^11^C] BU99008) [66,67]. A study with ([^11^C] BU99008) PET in PD patients showed increased reactive astrogliosis in early-stage PD patients at cortical areas and the midbrain [68], which decreased as the disease progressed. The authors also reported an association of astroglial activation in frontal cortices with global cognitive impairment.

### 2.5. Anti-Inflammatory Drugs in PD

Therefore, we would hypothesise that iron accumulation and neuroinflammation are intimately linked and that to mitigate the damaging effects of iron accumulation, it would be necessary to have a combination therapy approach with both iron chelators and anti-inflammatory drugs to remove the driver for such iron accumulation. Natural bio-agents, i.e., nutraceuticals, natural phytobioactive compounds, saffron, turmeric, resveratrol, and polygonum multiflorum have all shown promise as anti-inflammatory compounds in vitro in cell cultures and animal models of PD, but as of yet, there are no clinical trials reported [69]. Clinical trials are about to commence to investigate whether azathioprine may suppress the immune system in PD. Studies are also evaluating compounds that could interfere with cellular signalling cascades, e.g., p38, MEK1, and MEK2 protein kinases, and diminish the inflammatory pathway. Several of these inhibitors are effective in animal models of disease and are now under investigation for the treatment of inflammatory diseases [70]. Minocycline, a semisynthetic tetracycline-derived antibiotic, has been shown to exert neuroprotective effects on various experimental models of Parkinson’s disease (PD), but clinical studies have failed to confirm the neuroprotective effect of minocycline in PD. [71]. Non-steroid anti-inflammatory drugs (NSAIDs) were initially proposed to have a potential preventive agent for Parkinson’s disease, which was based on laboratory evidence as well as results from a few large observational studies [72,73,74,75], but this was not confirmed in later studies [76]. Such discrepancies may depend on whether the NSAIDs were administered regularly and for a long time [77].

Ferroptosis is a form of regulated cell death involving the iron-dependent formation of reactive free radicals, resulting in the generation of lipid hydroperoxides within membrane phospholipids rich in polyunsaturated fatty acids (PUFAs). Three factors are involved in ferroptotic cell death: increased free intracellular iron, oxidation of membrane PUFAs, and depletion of the glutathione/glutathione peroxidase system responsible for the reduction of lipid peroxides to lipid alcohols [78]. Recent studies involving both patients and animal models have suggested that ferroptosis is likely to be involved in a number of neurodegenerative diseases, including PD. During PD progression, there is an increase in oxidative stress, lipid peroxidation, and mitochondrial dysfunction associated with the depletion of antioxidant components of the glutathione system. It has been proposed that inhibitors of ferroptosis such as ferrostatin or iron chelators could play a therapeutic role in the treatment of PD [79].

## 3. Chelation Therapy

Iron chelation therapy was initially developed for patients with haemoglobinopathies, principally the thalassaemias and sickle cell diseases [80,81,82], whose treatment in the 1960s and 1970s involved repeated blood transfusions, compensating for the anaemia caused by the disease. However, this resulted in massive iron overload in the liver, spleen, endocrine tissues, and heart. Since each unit of transfused blood contains approximately 200 mg of iron, and humans cannot increase their iron excretion to compensate for iron loading, the therapy almost inevitably resulted in fatal heart failure by the age of 20. The incidence of heart failure was shown to be due to presence of highly toxic non-transferrin-bound iron (NTBI) released from the liver, which was responsible for the cardiac damage. The successful chelation of the excessive amounts of iron from these patients has transformed their lives. These same chelators have subsequently been used to mobilise iron from the pituitary gland and the brain [83], and more recently, there have been exciting developments in the synthesis of iron chelators for use in neurodegenerative diseases, all of which will be reviewed below.

### 3.1. Iron Chelators in Current Clinical Use

**Figure 3:**
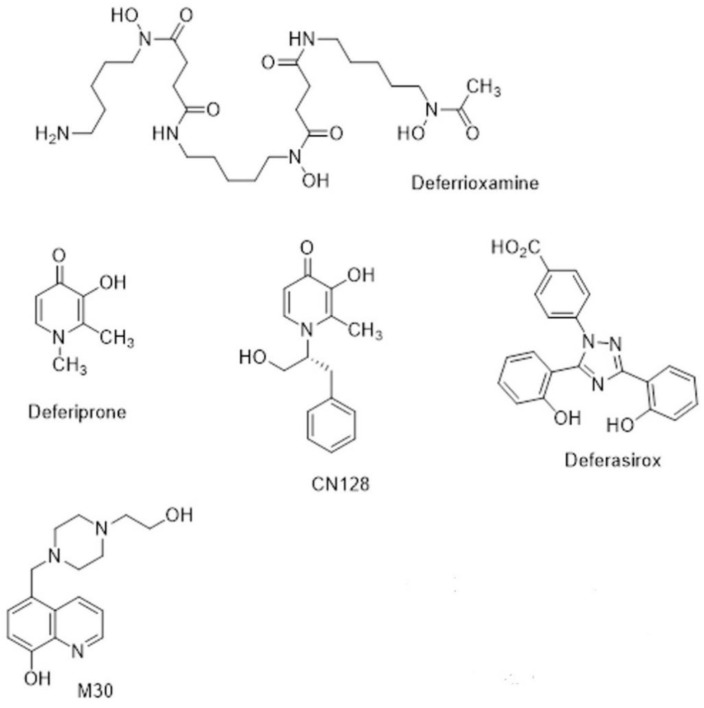
Chemical structures of chelators.

The naturally occurring siderophore, deferrioxamine (DFO) (Figure 3), contains three bidentate hydroxamate ligands and forms a very stable hexadentate complex with ferric iron. First introduced for transfusional iron overload in the early 1970s, DFO initially gave poor results because it was not orally active and had a short half-life (20–30 min). The development of continuous subcutaneous infusion of DFO by a portable pump [84] combined with sensible schedules for the optimal use of the pump [85] resulted in three independent studies demonstrating prolonged cardiac-disease-free survival in patents by the 1990s [86,87,88]. Unfortunately, patients who could not or would not comply developed cardiac failure or arrhythmia much more rapidly [89]. There was clearly a need for an orally active chelator, thereby improving compliance, which was as effective in iron chelation as DFO.

The small, lipophilic, bidentate chelator of the 3-hydroxypyridin-4-one family, deferiprone (DFP) (Figure 3), forms a stable 3:1 complex with Fe^3+^ and was introduced into clinical practice in the 1980s [90]. While it is orally active, its half-life is only 3–4 h, which means that it must be administered three times a day at doses of 75 mg/kg/day to maintain sufficient negative iron balance (50 mg/kg/day DFO) [91,92]. DFP has good bioavailability, but its clearance is accelerated by rapid biotransformation: approximately 85% of the drug is metabolised to a nonchelating 3-*O*-glucuronide conjugate [93]. There are important side effects of DFP, namely agranulocytosis and milder forms of neutropenia, which require appropriate clinical monitoring [94]. However, DFP enters cells and can access intracellular chelateable iron more readily than DFO [95]. DFP possesses cardioprotective effects [96], and a multi-centre comparison showed that DFP+DFO was more effective in removing cardiac iron than DFO and was superior in clearing hepatic iron than either DFO or DFP alone [97].

Subsequent to the advent of DFP, there was an upsurge in interest in other orally active chelators [98]. Desferrithiocin, a tridentate microbial chelator, was shown to be remarkably effective in mobilising hepatic iron in an iron-loaded rat model [99] and in iron-loaded monkeys [100,101]. However, its iron chelate, ferrithiocin, was nephrotoxic in animals [102], as were many of a series of synthetic derivatives of desferrithiocin [103]. Novartis initiated a massive research project that involved the evaluation of a vast number of chelators of different chemical classes, during which a completely new chemical class of tridentate iron chelators, the bis-hydroxyphenyltriazoles, was discovered. Forty derivatives of the triazole series were synthesised, and ultimately, ICL670 4-[(3,5-Bis-(2-hydroxyphenyl)-1,2,4) triazol-1-yl]-benzoic acid (Figure 3) was selected as a safe and effective oral chelator. ICL670, now deferasirox (DFX), not only showed high oral potency and good tolerability in animals [96] but also has a plasma half-life of 12–16 h, which means that when taken once per day, it effectively eliminated NTBI from the circulation [104]. DFX is as effective in clinical studies as DFO at doses of 20–30 mg/kg [105]. An extensive study involving 1744 patients demonstrated that fixed starting doses of DFX, based on transfusional iron intake, with dose titration guided by serum ferritin trends and safety markers provide clinically acceptable chelation in patients with transfusional iron overload from various types of anaemia [106].

### 3.2. Appication of Currently Available Chelators in Clinical Studies of PD

While there were early studies on AD patients using DFO administered intramuscularly [107], the first utilisation of an orally active chelator, DFP, was in patients with Friedreich’s ataxia [108] in which the mitochondrial iron chaperone, frataxin, is deficient. However, the first indications that iron chelation might be an effective therapeutic approach for PD came initially from studies on an animal model of PD in which we showed that the iron chelators, DFO and DFP, were able to reduce the iron content in various brain regions and to induce neuroprotection [109,110]. The beneficial effects of oral DFP were later demonstrated in two clinical trials on cohorts of PD patients [111,112]. In both studies, patients receiving 30 mg/kg/day showed decreases in SN iron content quantitated by MRI and improved UPDRS (Unified Parkinson’s Disease Rating Scale) scores (UPDRS evaluates various aspects of Parkinson’s disease including non-motor and motor experiences of daily living and motor complications). No adverse effect was observed on haematological parameters. In the study by Martin-Bastida et al. [112], chelation from other brain regions was also studied by MRI T2*, and it was established that there was selective iron removal from specific brain regions: first, after three months, from the caudate nucleus, then, after six months, from the dentate nucleus and to a lesser extent from the substantia nigra. Using R2* sequences, a decrease in substantia nigra iron was also observed in the study by Devos et al. [111] after six and twelve months. It was interesting that in the study by Martin-Bastida et al. [112], patients with high values of inflammatory markers such as IL-6 showed a poor response to iron chelation. This may indicate that once inflammation has occurred, blocking iron within the macrophages of the reticuloendothelial system, and perhaps also in the glial cells of the brain, chelators are no longer able to access previously potentially chelatable iron pools. A major inconvenience of the use of DFP is the incidence of agranulocytosis and neutropenia, which was observed in a few patients in both studies. While this undesirable side effect could be resolved by the cessation of the oral therapy, it nonetheless meant that all PD patients who entered these clinical studies were required to undergo weekly testing for white cell counts. This clearly not only complicates the logistics of clinical trials but also represents a major handicap in the potential use of this class of chelator for more extensive use on a larger population of patients. Further phase II clinical trials with DFP in early-stage PD patients are in course.

Thus, while these clinical studies clearly establish the therapeutic potential of chelation therapy in the long-term treatment of PD, there remain two outstanding issues to be resolved. Firstly, is it possible to design and develop hydroxypyridinone chelators that do not interfere with white cell counts, or alternatively to find other classes of iron chelators that do not have this potential disadvantage? This is briefly discussed in Section 3.3. Secondly, does long-term chelation therapy, even at the relatively low doses used in these studies, interfere with iron homeostasis in the brain, particularly in oligodendrocytes, which are involved in the myelination of neurons? Mature myelinating oligodendrocytes have the highest iron concentrations of all brain cells, and iron has been proposed to be involved in myelin production, not only as a necessary co-factor for cholesterol and lipid biosynthesis but also on account of its requirement for oxidative metabolism, which occurs in oligodendrocytes at a higher rate than other brain cells [113]. The answer to this question remains unknown.

### 3.3. Non-Invasive Evaluation of Brain Iron Chelation Therapy

Iron-sensitive magnetic resonance could potentially be considered as a monitoring tool for iron chelation therapies in PD as illustrated by two clinical trials with the iron cheater DFP [111,112]. The first study [111] showed iron removal in SN at 6 and 12 months and at a putaminal level only at 12 months after chelation when measured with R2* (the inverse of T2*), which in turn was associated with significant motor improvement throughout the study. In our study [112], we showed iron chelation at extranigral nuclei including caudate nucleus at the 3rd and 6th month and dentate nucleus after the 6th month after DFP treatment when measured with T2* imaging, which was also associated with a tendency of motor improvement in the short period of treatment. In our cohort, we found that patients with significant nigral iron removal displayed lower systemic ferritin levels at the end of the study, serum ferritin being indicative of an inflammatory process in our studies. This indicated, as pointed out earlier, the inability of chelators to be effective when there was an underlying inflammatory response.

It is worth mentioning that the use of high-pass filtered phase imaging and quantitative susceptibility mapping may be of therapeutic interest in further chelation studies. Relaxometry techniques (T2* and R2*) estimate iron deposition using gradient echo sequences sensitive to variations of transverse relaxation time between different tissues affected by magnetic field inhomogeneities [14]. Furthermore, relaxometry imaging is potentially affected by changes in tissue water content and background field inhomogeneities such as those resulting from air–tissue interfaces.

### 3.4. New Chelators for Brain Iron Chelation

During the last decade, new hydroxypyridinone chelators have been designed and tested that do not have the undesirable effects on white cell count in rodents and primates. This has led to the identification of a lead compound, CN128 (Figure 3), which combines the therapeutic properties of DFP (including BBB penetration) while lacking the induction of agranulocytosis and neutropenia when administered over a nine-month period in primates [114]. Further studies on CN128 in models of PD are ongoing [115].

With regard to alternative classes of iron chelators than the hydroxypyridinones, DFX, the other clinically approved orally active chelator, does not appear to be available for use in neurodegenerative diseases. Several other classes of iron chelator have been proposed, which will be discussed here.

Iron accumulation, ROS, and inflammation occur with ageing and in many age-related neurodegenerative diseases, such as Parkinson’s disease (PD). They are often accompanied by reduced activity of cytoprotective antioxidant defence mechanisms, including both enzymes that interact with some of the reactive oxidants, as well as several antioxidant molecules present within the cell. The removal of iron by the chelator will prevent the excessive formation of reactive oxygen species. This has led Youdim and collaborators to propose the use of multi-target drugs to induce disease modification, by slowing the process of neurodegeneration in Parkinson’s disease (PD) [116,117], as a better strategy. One example is M30 (Figure 3), which is an iron chelator-radical scavenging drug that has brain-selective monoamine oxidase (MAO) A/B inhibitor activity with neuroprotective and neurorestorative activities for nigrostriatal dopamine neurons in animal models of PD [116]. 

The dysfunctional homeostasis of iron and copper can provoke ROS-mediated oxidation, misfolding, and aggregation of certain proteins, such as a-synuclein in PD, and this is the rationale behind therapies based on chelation. However, in order to differentiate this approach from the bulk removal of metal ions using strong systemic chelation therapy, as described earlier for the thalassemias, the term Metal–Protein Attenuating Compounds (MPAC) was used to describe moderate chelators that are able to disrupt specific (i.e., targeted), abnormal metal–protein interactions [118]. Several MPACs have been developed that aim at disrupting specific, abnormal metal–protein interactions. These weaker chelators should stabilise the abnormal concentrations of the metal ions, without inflicting damage to other processes that require their presence. PBT434 is a novel quinazolinone compound with a moderate affinity metal-binding motif, which is in development for use in Parkinsonian conditions. In vitro, PBT434 was far less potent than deferiprone or deferoxamine at lowering cellular iron levels yet was found to inhibit iron-mediated redox activity and iron-mediated aggregation of α-synuclein [119]. The N-acylhydrazones represent a class of promising MPACs for the management of metal-enhanced aggregopathies. 1-methyl-1H-imidazole-2-carboxaldehyde isonicotinoyl hydrazone (X1INH) attenuates abnormal copper(I)/copper (II)-α-Syn interactions and affects protein aggregation in a cellular model of synucleinopathy [120].

In recent years, there has been a growing interest in naturally occurring phytochemicals, which have gained a lot of attention as a potential therapeutic agent for preventing neurodegeneration [121]. Many reports have shown that flavonoids, a large family of plant-derived hydroxylated polyphenols, have anti-inflammatory and antioxidant properties, are able to modulate protein misfolding as well as having effects such as antineoplastic and anti-obesity actions. In particular, it is well established that the long-term consumption of the green tea-derived flavonoids catechin and epigallocatechin gallate (EGCG) can attenuate the onset of PD [122,123]. Other naturally occurring compounds, such as phenylpropanoids, quinones, saponins, alkaloids, and terpenoids compounds have well recognised anti-oxidative and anti-inflammatory activities in addition to inhibitory roles with respect to protein misfolding and the regulatory effects on specific PD-related pathways reviewed in [124]. Further in vivo studies are needed to ascertain whether such compounds can cross the BBB and retain their anti-PD activity,

## 4. Concluding Remarks

The underlying characteristics of Parkinson’s disease include neuroinflammation, the generation of ROS, and localised iron accumulation, particularly in the SN. The combined effects of inflammation on iron metabolism in glial cells and their toxic consequences on neurons suggest that therapeutic approaches that target the regulation of glial function may represent a promising approach to the treatment of iron-mediated neurodegenerative diseases, such as PD. Neuroinflammation would stimulate the classic iron-withholding response seen in the anaemia of chronic disease, which is reflected in the poor response to iron chelation therapy observed in our clinical trial [109] among PD patients with inflammation. This would also be in agreement with the observation that the long-term utilisation of non-steroid anti-inflammatory drugs protects from PD. Thus, a combined approach involving multi-target drugs, combining iron chelation, antioxidant, and anti-inflammatory properties may represent the way forward.

## Data Availability

Data presented in this study are openly available in the quoted referenced journals.
